# A Retrospective Study of the Clinical Burden of Hospitalized All-Cause and Pneumococcal Pneumonia in Canada

**DOI:** 10.1155/2016/3605834

**Published:** 2016-03-29

**Authors:** Shelly A. McNeil, Nawab Qizilbash, Jian Ye, Sharon Gray, Giovanni Zanotti, Samantha Munson, Nathalie Dartois, Craig Laferriere

**Affiliations:** ^1^Canadian Center for Vaccinology, IWK Health Centre and Nova Scotia Health Authority, Dalhousie University, Halifax, NS, Canada B3K 6R8; ^2^OXON Epidemiology Ltd., London NW1 2FD, UK; ^3^London School of Hygiene & Tropical Medicine, London WC1E 7HT, UK; ^4^Pfizer Inc., Collegeville, PA 19426, USA; ^5^Pfizer Canada, Kirkland, QC, Canada H9J 2M5; ^6^Pfizer, 75668 Paris Cedex 14, France

## Abstract

*Background*. Routine vaccination against* Streptococcus pneumoniae* is recommended in Canada for infants, the elderly, and individuals with chronic comorbidity. National incidence and burden of all-cause and pneumococcal pneumonia in Canada (excluding Quebec) were assessed.* Methods*. Incidence, length of stay, and case-fatality rates of hospitalized all-cause and pneumococcal pneumonia were determined for 2004–2010 using ICD-10 discharge data from the Canadian Institutes for Health Information Discharge Abstract Database. Population-at-risk data were obtained from the Statistics Canada census. Temporal changes in pneumococcal and all-cause pneumonia rates in adults ≥65 years were analyzed by logistic regression.* Results*. Hospitalization for all-cause pneumonia was highest in children <5 years and in adults >70 years and declined significantly from 1766/100,000 to 1537/100,000 per year in individuals aged ≥65 years (*P* < 0.001). Overall hospitalization for pneumococcal pneumonia also declined from 6.40/100,000 to 5.08/100,000 per year. Case-fatality rates were stable (11.6% to 12.3%). Elderly individuals had longer length of stay and higher case-fatality rates than younger groups.* Conclusions*. All-cause and pneumococcal pneumonia hospitalization rates declined between 2004 and 2010 in Canada (excluding Quebec). Direct and indirect effects from pediatric pneumococcal immunization may partly explain some of this decline. Nevertheless, the burden of disease from pneumonia remains high.

## 1. Introduction

*Streptococcus pneumoniae* is an important cause of pneumonia and invasive pneumococcal diseases (IPDs) which are leading causes of morbidity and mortality, particularly in infants and older adults [1, 2]. Before routine pneumococcal vaccination in Canada, the burden of pneumococcal disease was substantial among all age groups. In 2001, an estimated 565,077 cases of pneumococcal disease in Canada resulted in 3002 deaths, mainly due to pneumonia in the elderly [3]. This clinical burden was associated with the loss of 54,330 life-years, and pneumococcal disease incurred an estimated cost to society of $193 million (Canadian dollars); IPD represented 17% of these overall costs [3].

In 2002, the Canadian National Advisory Committee on Immunization (NACI) first recommended 4 doses of 7-valent pneumococcal conjugate vaccine (PCV7) in routine infant immunization schedules for all children aged <2 years and children aged 2–5 years at high risk for pneumococcal infection [4]. Vaccination with PCV7 was implemented in all provinces and territories by 2006 [5]. The programs resulted in dramatic decreases in IPD and isolation of antibiotic-resistant PCV serotypes, more than halving the rates of IPD in children [3, 6, 7].

Although a herd effect has been observed for IPD in Canadian adults [8–10], no such effect has been reported for pneumonia. This is in contrast with the United States, where a herd effect has been demonstrated for the two conditions [11–13].

Healthcare policy makers rely on availability of accurate data on the burden of illness within their region. This paper reports the incidence and case-fatality rates of hospitalized all-cause and pneumococcal pneumonia, and mean length of stay (LOS) of hospitalization for all ages across Canada (excluding Quebec) between 2004 and 2010.

## 2. Methods

### 2.1. Study Objectives

The primary objective of this study was to quantify the incidence rate of all-cause infectious pneumonia using retrospective data for patients admitted to all hospitals across all provinces and territories in Canada during the study period from 2004 to 2010. Secondary objectives included quantifying the length of hospital stay and the mortality rate associated with all-cause pneumonia and the incidence rate of pneumococcal pneumonia.

### 2.2. Data Collection

Data were obtained from the Canadian Institute for Health Information (CIHI) Discharge Abstract Database (DAD) [14], which contains demographic, administrative, and clinical data for patients admitted to all hospitals in Canada, except Quebec. Since 2004-2005, all DAD records have been reported using the International Classification of Diseases, Tenth Revision, Canadian Enhancement (ICD-10-CA) codes [15]. Data were collected at each hospital from patient administrative and clinical record systems and were compiled each financial year ending 31 March. Hospitalization in Quebec was not included in the analysis because ICD-9 diagnosis codes were used in this province during part of the study period.

Data collected included patient demographics, hospitalization information (including the ICD-10-CA codes for the most responsible diagnosis and month and year of hospitalization), in-hospital case-fatality, hospital length of stay (LOS), and province/territory. Cases of hospitalization due to all-cause pneumonia and pneumococcal pneumonia were identified using the corresponding ICD-10-CA codes (Supplemental Table  1 in Supplementary Material available online at http://dx.doi.org/10.1155/2016/3605834). Population-at-risk data were obtained from Statistics Canada census information [16]. Data were provided in an anonymous and aggregate fashion, and ethics approval was not required.

### 2.3. Data Analyses

Hospitalization data for patients admitted to all hospitals across all provinces and territories in Canada except Quebec (2004–2010) and population at-risk data obtained from Statistics Canada census were used to calculate incidence rate. The incidence per 100,000 persons per year and 95% confidence intervals (CIs) of hospitalization for each condition were calculated based on Poisson distribution. Case-fatality rates (95% CIs) were calculated as the number of deaths divided by the corresponding number of hospitalized cases multiplied by 100. LOS was calculated as the number of days in the hospital and reported as mean with standard deviations (SD). Patient subgroups were defined by age, gender, and province/territory. Cases without sex or age information were excluded. The incidence rates of pneumococcal and all-cause pneumonia in adults aged ≥65 years were investigated by logistic regression analyses using year as the independent variable. All analyses were performed using SAS software (SAS Institute, Cary, NC; version 9.1.3 and version 9.2).

## 3. Results

Between 2004 and 2010, hospitalized cases meeting study criteria ranged from 88,151 (2004-2005) to 89,785 (2009-2010) for all-cause pneumonia and 1563 (2004-2005) to 1315 (2009-2010) for pneumococcal pneumonia.

### 3.1. All-Cause Pneumonia

**Figure 1:**
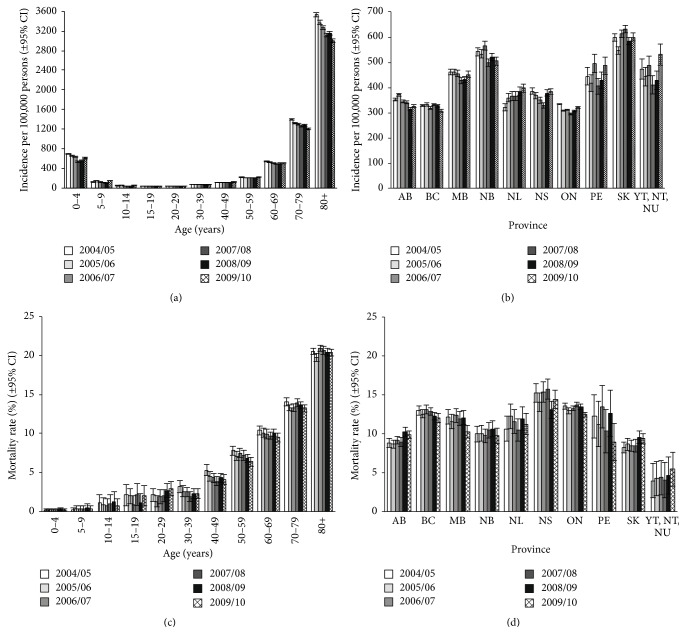
Annual incidence of hospitalization in Canada from 2004-2005 to 2009-2010 due to all-cause pneumonia by age (a) and province (b); and annual case-fatality rate due to all-cause pneumonia by age (c) and province (d). Error bars indicate 95% confidence intervals. AB, Alberta; BC, British Columbia; MB, Manitoba; NB, New Brunswick; NL, Newfoundland and Labrador; NS, Nova Scotia; NT, Northwest Territories; NU, Nunavut; PE, Prince Edward Island; SK, Saskatchewan; YT, Yukon.

**Figure 2:**
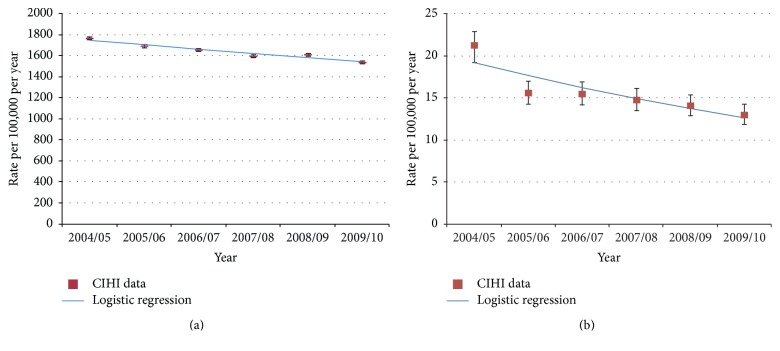
Logistic regression analysis in Canada from 2004-2005 to 2009-2010 in individuals aged ≥65 years for (a) all-cause pneumonia and (b) pneumococcal pneumonia. Error bars indicate 95% confidence intervals. CIHI, Canadian Institute for Health Information.

Children aged <5 years and adults aged >70 years had the highest incidence of hospitalized all-cause pneumonia (Figure 1(a) and Supplemental Table  2). In adults, incidence increased sharply after the age of 60 years, with markedly higher incidences of pneumonia-related hospitalization versus all younger age groups in all years (Figure 1(a) and Table 1). An overall decline in the incidence of hospitalization due to all-cause pneumonia was observed over the study period owing primarily to decreases in the incidence among the youngest children and adults aged ≥65 years. In the <4-year-olds group, incidence fell from 694 (95% CI: 680–708) in 2004-2005 to 611 (95% CI: 598–624) in 2009-2010, and in the ≥65-year-olds group incidence fell from 1766 (95% CI: 1751–1780) to 1537 (95% CI: 1524–1550) during the same period. In other age groups, the rates of all-cause pneumonia were lower but did not decline over the study period (Figure 1(a)). Logistic regression was used to demonstrate that the decline observed in adults aged ≥65 years (13%) was statistically significant (*P* < 0.001, Figure 2(a)). In all study years, the incidence of all-cause pneumonia was higher in males than females (Table 1). The frequencies for hospital admissions due to all-cause pneumonia for each province or territory are presented in Figure 1(b). The mean LOS for hospitalization for all-cause pneumonia remained stable over time in the overall population (9.99 days in 2004-2005 and 10.54 days in 2009-2010) (Table 1) and increased with increasing age.

Overall case-fatality rates associated with all-cause pneumonia were relatively stable over the study period, ranging from 11.6% in 2009-2010 to 12.3% in 2004-2005 (Table 1). Mortality increased with increasing age (Figure 1(c) and Supplemental Table  2) and was the highest in those aged ≥80 years (19.7%–20.9%). In patients aged ≥65 years, the 2009-2010 case-fatality rate for hospitalized all-cause pneumonia was 16.7%, whereas it was ≤1% in children aged <10 years (Table 1). Case-fatality rates varied by province but were relatively stable within each province (Figure 1(d)). In most study years, case-fatality rates were higher in males than females (Table 1).

### 3.2. Pneumococcal Pneumonia

**Figure 3:**
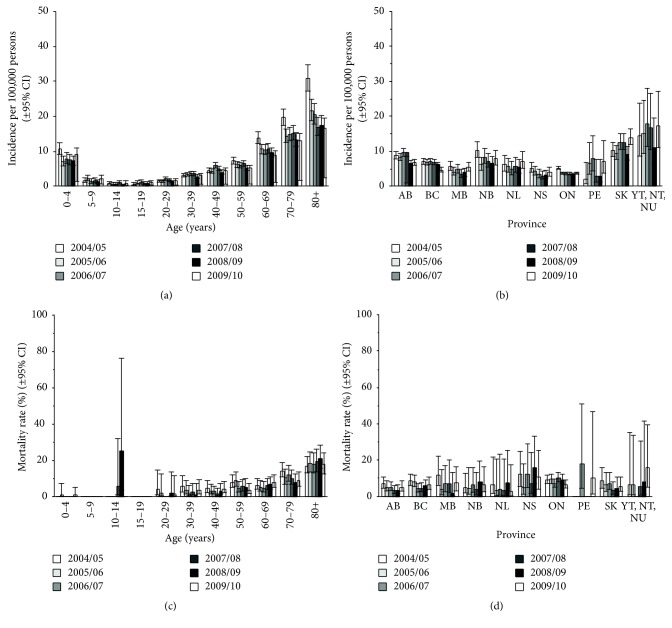
Annual incidence of hospitalization in Canada from 2004-2005 to 2009-2010 due to pneumococcal pneumonia by age (a) and province (b); and annual case-fatality rate due to pneumococcal pneumonia by age (c) and province (d). Note that case-fatality rates were 0 in PE in 2005-2006, 2007-2008, and 2009-2010 and in YT, NT, and NU in 2006-2007. Error bars indicate 95% confidence intervals. AB, Alberta; BC, British Columbia; MB, Manitoba; NB, New Brunswick; NL, Newfoundland and Labrador; NS, Nova Scotia; NT, Northwest Territories; NU, Nunavut; PE, Prince Edward Island; SK, Saskatchewan; YT, Yukon.

The highest incidence of hospitalization due to pneumococcal pneumonia occurred in children aged <5 years (10.64/100,000 [95% CI: 9.02–12.54] in 2004-2005 and 9.14/100,000 [95% CI: 7.70–10.86] in 2009-2010) and in adults aged ≥65 years (21.27/100,000 [95% CI: 19.27–22.95] in 2004-2005 and 13.00/100,000 [95% CI: 11.86–14.25] in 2009-2010) (Table 2 and Supplemental Table  3). As with all-cause hospitalized pneumonia, the incidence curve by age was J-shaped, with rates in adults increasing with increasing age (Figure 3(a)).

Across all ages, the annual incidence of hospitalization for pneumococcal pneumonia declined from 6.40/100,000 (95% CI: 6.09–6.73) in 2004-2005 to 5.08/100,000 (95% CI: 4.81–5.36) in 2009-2010 (Table 2). Declines were seen in the 0–4-year-olds group from 10.64/100,000 (95% CI: 9.02–12.54) in 2004-2005 to 7.21/100,000 (95% CI: 5.93–8.76) in 2008-2009. Declines were also seen in age groups >50 years. Of note, the 39% decline seen in adults aged ≥65 years (21.27/100,000 [95% CI: 19.72–22.95] in 2004-2005 to 13.00/100,000 [95% CI: 11.86–14.25] in 2009-2010) was statistically significant (*P* < 0.001, Figure 2(b)). The annual incidence of hospitalized pneumococcal disease was the highest in the Canadian North (Northwest Territories, Yukon, and Nunavut), with rates 2−4-fold higher than those in Ontario and Nova Scotia (Figure 3(b)).

The mean LOS for hospitalization due to pneumococcal pneumonia remained stable in the total population over time (11.93 days in 2004-2005 and 11.31 days in 2009-2010; Table 2). Older patients tended to have longer LOS than younger patients. In the overall study population, case-fatality rates for pneumococcal pneumonia remained relatively stable over the study period from 8.6% (95% CI: 7.3%–10.1%) in 2004-2005 to 6.4% (95% CI: 5.2%–7.8%) in 2009-2010 (Table 2). A high case-fatality rate was observed among children aged 10–14 years in 2008-2009 (Figure 3(c) and Supplemental Table  3); however, this reflected a single death among only 4 cases. Generally, an increase in case-fatality with increasing age was observed, rising sharply in those aged ≥60 years (Table 2). Case-fatality rates varied greatly by province and were highly variable by year within a province (Figure 3(d)).

## 4. Discussion

From 2004 to 2010, an overall decrease in the incidence of hospitalization for all-cause and pneumococcal pneumonia was observed in Canada in this study. The highest burden of pneumonia leading to hospitalization—all-cause and pneumococcal—was seen in the very young (children aged <5 years) and in older adults, particularly those aged ≥65 years. The risk of mortality associated with pneumonia increased with age in older adults. Concomitant with these age-related trends, the mean LOS for all-cause and pneumococcal pneumonia hospital admissions notably increased with advancing age.

The temporal trends in pneumococcal pneumonia incidence observed in several provinces (Figure 3(b)) are similar to those previously reported for IPD from provincial surveillance programs. For example, overall IPD declined in British Columbia from 2008 through 2010 [10], whereas a transient increase in IPD was observed in Alberta in 2006-2007, followed by a steep decline in 2009-2010 [8]. Overall rates of IPD were not reported for Ontario in a recent publication [9] but the decline of IPD in children aged <5 years between 2002 and 2006 may be reflected in the decline in pneumococcal pneumonia seen in Figure 3(b) between 2004-2005 and 2005-2006 for this province.

The case-fatality rate due to IPD in Ontarian adults aged ≥65 years was reported to decline from 29.5% to 25.7% between 1995 and 2011 (*P* < 0.005) [9], but the data shown in Figure 3(d) for mortality from pneumococcal pneumonia in Ontario does not reflect this, possibly because of the shorter time span and variability of the data.

The 39% decline in pneumococcal pneumonia and 13% decline in all-cause pneumonia in adults aged ≥65 years between 2004 and 2010 are significant, corresponding to improvements in health measures and warrant attribution. Simonsen and colleagues found a 54% decline in nonbacteremic pneumococcal pneumonia in US adults aged ≥65 years between baseline 1996–1999 and the 2005-2006 seasons [11]. By using differences in vaccination rates between states, they were able to demonstrate that PCV7 had direct and indirect effects in reducing both IPD and pneumococcal pneumonia in children and adults [11]. Such an approach could not be used in Canada even though publicly funded PCV7 programs were initiated in different provinces at different times between 2002 and 2005 [17]. Active surveillance in Ontario showed that IPD in children <5 years old began to decline as early as 2002, before the publicly funded program began in 2005 [9]. Private sales were estimated at 1 dose per child in the birth cohort during this time [9]. Private sales and lack of information on coverage rates make it difficult to apply the same methodology to demonstrate that the reduction of pneumonia was due to indirect effects in Canada.

Reduction of vaccine serotype IPD in Canadian adults ≥65 years old has been observed from surveillance data in Alberta, British Columbia, Ontario, and Quebec [8–10, 18]; however, serotype replacement occurred in Ontario and Quebec to the extent that there has not been a decline in the overall rate of IPD [9, 18]. In a Quebec study of hospital admissions for pneumonia, in contrast to reductions seen in the children <5 years old after the introduction of a catch-up program with PCV7 in December 2004 [19], there was no reduction in hospitalization for pneumonia in adults aged ≥65 years between 1996 and 2007 [20]. A decline in admissions was noted in younger age groups in 1999, which was attributed to changes in infectious diseases guidelines published in 1998 [20].

The present data, together with previously published reports [5], are consistent with the assertion that vaccination programs in Canada have had an impact on the incidence of pneumococcal disease, particularly among children aged <4 years and adults aged ≥65 years. However, disease incidence and associated health-service utilization remain high, with little change in hospital LOS or case-fatality rates over time, suggesting a continuing need to improve the uptake and coverage of available vaccines in older adults and at-risk groups and to continue development programs that offer the prospect of new higher-valent vaccines targeting additional serotypes.

The decline in the rate of all-cause pneumonia we report was 27 times greater than the decline in pneumococcal pneumonia (−229 compared to −8.27 cases per 100,000 per year). Even though nonbacteremic pneumococcal pneumonia may go undiagnosed, the incidence rate is estimated to be only 3 to 5 times higher than invasive disease [21]. Thus, it is likely that other factors accounted for the decline in all-cause pneumonia in adults aged ≥65 years. Other factors hypothetically contributing to the decline of all-cause pneumonia are tabulated in Table 3. Only the last item in the list, the Canadian Thoracic Society guidelines published in 2003 for the treatment of acute exacerbation of chronic bronchitis [22], appears timed to explain the decrease in all-cause pneumonia. Canada was the first country to adopt these guidelines targeting patients with greatest risk for a poor outcome and who were likely to benefit most from early antibiotic treatment [23].

The importance of chronic pulmonary disease in pneumococcal pneumonia was demonstrated in a recent efficacy study of a 13-valent pneumococcal conjugate vaccine against pneumococcal community-acquired pneumonia (CAP) in adults aged ≥65 years [31]. While 10.2% of the 84,496 subjects recruited into this study self-reported with chronic pulmonary disease [31], 59% of the 139 subjects diagnosed with vaccine-type pneumococcal pneumonia had physician-diagnosed chronic pulmonary disease (Pfizer Inc.; data on file). Likewise, other high risk groups such as diabetics, persons with heart disease, and persons with multiple comorbidities made up the majority of subjects suffering from pneumococcal vaccine-type CAP.

It is likely that both the indirect effects from the pediatric PCV7 program and the early treatment of patients with acute exacerbation of chronic obstructive pulmonary disease helped to reduce the rate of all-cause pneumonia in Canada during the time period in this study, but there is insufficient information to be able to quantify each contribution.

This study has several limitations. Sample sizes were small in some provinces/territories because of small populations. Variations in incidence and case-fatality rates by province may reflect differences in practices (e.g., only very sick patients may be hospitalized in some provinces). Cases treated as outpatients were not included, leading to probable underreporting of incidence. Although a major strength of the study is that the DAD provides comprehensive data on hospitalization and the census population data provide an appropriate denominator to calculate hospitalized incidence rates, data were derived from ICD-10-CA coding based on physician notes, the quality of which is not readily available at the CIHI. The incidence of hospitalization due to pneumococcal pneumonia was 4.77–6.40/100,000 versus 336–361/100,000 for all-cause pneumonia. Given that approximately 30% of all-cause pneumonia may be pneumococcal pneumonia [21], it is likely that a specific diagnosis of pneumococcal pneumonia is underreported. This is not surprising given that a specific etiology of hospitalized pneumonia is rarely identified except in cases of IPD. Although the diagnosis of pneumococcal pneumonia by ICD-10-CA coding has relatively low sensitivity, it is likely to be highly specific (i.e., cases assigned this code are very likely to be true cases of pneumonia related to* S. pneumonia*). This method for identifying cases is influenced not only by clinical coding practices, but also by changes in microbiologic testing practices, such as increased use of pneumococcal urinary antigen testing [32]. Microbial etiology was established for 67% of 184 patients presenting with CAP when a PCR assay was used, versus 60% with conventional methods [33], and* S. pneumoniae* was the leading causative agent. Any of these factors could lead to an underestimation of pneumococcal pneumonia cases. However, the incidence of pneumococcal pneumonia for adults aged ≥65 years in 2009-2010 in the current study (13/100,000) is similar to that seen for IPD in 2010 in Alberta [8] and British Columbia [10] and slightly lower than that in Ontario [9].

The exclusion of influenza-related codes, such as J11.0 (influenza with pneumonia, virus not identified), means that not all cases of hospitalization linked to pneumonia were included within the all-cause or pneumococcal pneumonia category. Data on comorbidities and concomitant risk factors for pneumonia were not included in this analysis. Respiratory viruses are frequently found in mixed infections with* S. pneumoniae* [33]. Finally, a more complete picture of vaccine impact and potential serotype replacement would emerge if serotype data were available. Although serotype 19A proportionally increased in Canada in the post-PCV7 era, the effect of PCV13 vaccination remains unknown [34]. In areas with <70% vaccine coverage, data showing increases in non-PCV serotypes should be interpreted cautiously [35].

## 5. Conclusions

In this retrospective burden of disease study, the incidence of all-cause and pneumococcal pneumonia requiring hospitalization decreased between 2004 and 2010 across Canadian provinces in the total population and in all age groups analyzed. Case-fatality rates associated with hospitalized all-cause and pneumococcal pneumonia were age-related, with increasing age associated with greater mortality. Despite the benefits of national vaccination programs, the burden of all-cause and pneumococcal disease in Canada remains high, particularly in younger children and older adults.

## Supplementary Material

Supplementary material includes a description of the ICD-10-CA codes for all-cause and pneumococcal pneumonia, and tables presenting the annual incidence and case-fatality rates for all-cause and pneumococcal pneumonia by age group.

## Figures and Tables

**Table 1:** Annual incidence, LOS, and case-fatality rates for hospitalization due to all-cause pneumonia in Canada, from 2004-2005 to 2009-2010.

	2004-2005	2005-2006	2006-2007	2007-2008	2008-2009	2009-2010
*Annual incidence*						
Number of cases/at-risk population	88151/24404747	87050/24663298	86679/24944522	84909/25242310	86839/25564644	89785/25891827
Incidence per 100,000 persons (95% CI)						
Overall	361 (359, 364)	353 (351, 355)	347 (345, 350)	336 (334, 339)	340 (337, 342)	347 (345, 349)
≥65 years of age	1766 (1751, 1780)	1692 (1678, 1707)	1655 (1641, 1669)	1595 (1582, 1609)	1611 (1598, 1624)	1537 (1524, 1550)
Males	382 (379, 386)	371 (367, 374)	367 (364, 371)	356 (353, 359)	355 (352, 358)	362 (359, 365)
Females	341 (337, 344)	336 (332, 339)	328 (325, 331)	317 (314, 320)	324 (321, 328)	332 (329, 335)
LOS, mean days ± SD						
Overall	9.99 ± 16.66	9.92 ± 15.51	10.30 ± 17.42	10.67 ± 18.73	11.03 ± 18.90	10.54 ± 20.09
0–17 years of age	3.90 ± 6.51	3.89 ± 8.12	3.93 ± 6.16	3.88 ± 6.35	3.96 ± 7.03	3.90 ± 8.10
18–69 years of age	9.13 ± 14.94	8.77 ± 14.5	9.07 ± 15.91	9.41 ± 19.10	9.64 ± 16.46	9.35 ± 17.32
≥70 years of age	11.98 ± 18.77	12.11 ± 16.95	12.54 ± 19.50	12.87 ± 19.84	13.30 ± 21.31	12.98 ± 23.18
*Case-fatality*						
Number of deaths/number of cases	10822/88151	10151/87050	10539/86679	10397/84909	10707/86839	10419/89785
% (95% CI)						
Overall	12.3 (12.1, 12.5)	11.7 (11.4, 11.9)	12.2 (11.9, 12.4)	12.2 (12.0, 12.5)	12.3 (12.1, 12.6)	11.6 (11.4, 11.8)
≥65 years of age	17.1 (16.8, 17.4)	16.4 (16.1, 16.8)	17.1 (16.7, 17.4)	17.1 (16.8, 17.5)	17.0 (16.7, 17.3)	16.7 (16.4, 17.0)
Males	12.8 (12.5, 13.1)	11.9 (11.6, 12.2)	12.5 (12.2, 12.8)	12.6 (12.3, 12.9)	12.9 (12.6, 13.3)	11.9 (11.6, 12.1)
Females	11.7 (11.4, 12.0)	11.4 (11.1, 11.7)	11.7 (11.4, 12.1)	11.8 (11.5, 12.2)	11.7 (11.4, 12.0)	11.3 (11.0, 11.6)

CI, confidence interval; LOS, length of stay; SD, standard deviation.

**Table 2:** Annual incidence, LOS, and case-fatality rates for hospitalization due to pneumococcal pneumonia in Canada, from 2004-2005 to 2009-2010.

	2004-2005	2005-2006	2006-2007	2007-2008	2008-2009	2009-2010
*Annual incidence*						
Number of cases/at-risk population	1563/24404747	1307/24663298	1420/24944522	1395/25242310	1219/25564644	1315/25891827
Incidence per 100,000 persons (95% CI)						
Overall	6.40 (6.09, 6.73)	5.30 (5.02, 5.59)	5.69 (5.40, 6.00)	5.53 (5.24, 5.82)	4.77 (4.51, 5.04)	5.08 (4.81, 5.36)
≥65 years of age	21.27 (19.72, 22.95)	15.61 (14.30, 17.04)	15.49 (14.20, 16.90)	14.78 (13.53, 16.14)	14.11 (12.90, 15.42)	13.00 (11.86, 14.25)
Males	7.34 (6.87, 7.84)	6.03 (5.61, 6.48)	6.73 (6.29, 7.21)	6.53 (6.09, 6.99)	5.66 (5.26, 6.09)	5.70 (5.30, 6.13)
Females	5.49 (5.09, 5.92)	4.58 (4.22, 4.98)	4.67 (4.31, 5.06)	4.54 (4.19, 4.93)	3.89 (3.56, 4.24)	4.47 (4.12, 4.85)
LOS, mean days ± SD						
Overall	11.93 ± 17.18	11.07 ± 18.53	11.37 ± 18.51	11.54 ± 17.42	12.16 ± 17.16	11.31 ± 18.13
0–17 years of age	5.96 ± 8.93	6.45 ± 10.18	6.35 ± 7.20	5.76 ± 6.54	10.36 ± 18.85	10.18 ± 31.26
18–69 years of age	12.24 ± 17.47	10.56 ± 14.56	11.75 ± 21.32	11.10 ± 17.26	11.75 ± 14.86	10.59 ± 13.22
≥70 years of age	13.53 ± 18.46	13.80 ± 26.01	12.47 ± 14.05	14.87 ± 19.92	13.62 ± 20.49	13.43 ± 18.00
*Case-fatality*						
Number of deaths/number of cases	134/1563	95/1307	87/1420	86/1395	83/1219	84/1315
% (95% CI)						
Overall	8.6 (7.3, 10.1)	7.3 (6.0, 8.8)	6.1 (5.0, 7.5)	6.2 (5.0, 7.6)	6.8 (5.5, 8.4)	6.4 (5.2, 7.8)
≥65 years of age	13.7 (11.3, 16.5)	12.7 (10.0, 15.9)	12.7 (10.0, 15.9)	12.2 (9.6, 15.4)	11.8 (9.2, 15.0)	12.0 (9.4, 15.4)
Males	8.9 (7.2, 11.0)	7.6 (5.9, 9.7)	6.1 (4.7, 8.0)	6.5 (5.0, 8.4)	6.4 (4.8, 8.4)	5.6 (4.2, 7.5)
Females	8.1 (6.3, 10.5)	6.8 (5.0, 9.2)	6.1 (4.5, 8.4)	5.7 (4.1, 7.9)	7.4 (5.4, 10.0)	7.4 (5.5, 9.8)

CI, confidence interval; LOS, length of stay; SD, standard deviation.

**Table 3:** Summary of practices and policy changes that could account for declining pneumonia rates in Canadianadults ≥65 years old.

Factor	Can this explain the decline in all-cause pneumonia between 2004 and 2010 in adults aged ≥65 years?	Reference
Changes in ICD-10 coding practices	No. Would expect changes in all ages groups	NA

Increased use of fluoroquinolones for lower respiratory tract infection	Maybe. Fluoroquinolone scripts were up in 2004-2005 but down again in 2008-2009	[24, 25]

Smoking amongst adults aged ≥65 years	No. Smoking remained constant at approximately 11%	Compare [26] with [27]

Pneumovax vaccination	No. PPSV23 immunization rates in adults aged ≥65 years were stable between 2006 and 2010 at approximately 38%	[28]

Influenza vaccination	No. Influenza immunization declined from 69.9% in 2006 to 52.8% in 2010 in adults aged ≥65 years	[28]

New clinical pathway for treatment in long-term care facilities, rather than in the hospital	No. Decline began before publication of protocol	[29]

Change in Infectious Disease Society of America Guidelines for the management of community-acquired pneumonia	No. Decline began before publication of guidelines	[30]

New guidelines for the treatment of acute exacerbations of chronic bronchitis	Yes. Focused antibiotic treatment of Groups 2 and 3 AECOPD published in 2003	[22]

AECOPD: acute exacerbations of chronic obstructive pulmonary disease; ICD: International Classification of Diseases; NA: not applicable; PPSV23: 23-valent pneumococcal polysaccharide vaccine.

## Abbreviations

CAD: Canadian dollars
CAP: Community-acquired pneumonia
CI: Confidence interval
CIHI: Canadian Institute for Health Information
DAD: Discharge Abstract Database
ICD-10-CM: International Classification of Diseases, Tenth Revision, Canadian Modification
IPD: Invasive pneumococcal disease
LOS: Length of stay
PCV: Pneumococcal conjugate vaccine
PPSV23: 23-valent pneumococcal polysaccharide vaccine.
