# Supplementary material for: A Retrospective Study of the Clinical Burden of Hospitalized All-Cause and Pneumococcal Pneumonia in Canada
Source: Can Respir J. 2016 Mar 29;2016:3605834. doi: 10.1155/2016/3605834 (PMC4904510; doi:10.1155/2016/3605834)
Supplement: Supplementary file 1 — Supplementary material includes a description of the ICD-10-CA codes for all-cause and pneumococcal pneumonia, and tables presenting the annual incidence and case-fatality rates for all-cause and pneumococcal pneumonia by age group. [file 3605834.f1.pdf]

Table S1. ICD-10 CA Codes

**All-Cause Pneumonia and Pneumococcal Pneumonia**	
J12.0–2, J12.8, J12.9	Viral pneumonia, not elsewhere classified: J12.0 (Adenoviral pneumonia); J12.1 (Respiratory syncytial virus pneumonia); J12.2 (Parainfluenza virus pneumonia); J12.8 (Other viral pneumonia), J12.9 (Viral pneumonia unspecified)
J13	Pneumonia due to *Streptococcus pneumoniae*
J14	Pneumonia due to *Haemophilus influenza*
J15.0–9	Bacterial pneumonia, not elsewhere classified: J15.0 (Pneumonia due to Klebsiella pneumoniae); J15.1 (Pneumonia due to Pseudomonas); J15.2 (Pneumonia due to Staphylococcus); J15.3 (Pneumonia due to Streptococcus, group B); J15.4 (Pneumonia due to other streptococci); J15.5 (Pneumonia due to Escherichia coli); J15.6 (Pneumonia due to other aerobic Gram-negative bacteria); J15.7 (Pneumonia due to Mycoplasma pneumoniae); J15.8 (Other bacterial pneumonia); J15.9 (Bacterial pneumonia, unspecified)
J16.0, J16.8	Pneumonia due to other infectious organisms, not elsewhere classified: J16.0 (Chlamydia pneumonia); J16.8 (Pneumonia due to other specified infectious organisms)
J17.0–3, J17.8	Pneumonia in diseases classified elsewhere: J17.0 (Pneumonia in diseases classified elsewhere); J17.1 (Pneumonia in viral diseases classified elsewhere); J17.2 (Pneumonia in mycoses); J17.3 (Pneumonia in parasitic diseases); J17.8 (Pneumonia in other diseases classified elsewhere)
J18.0–2, J18.8, J18.9	Pneumonia, organism unspecified: J18.0 (Bronchopneumonia, unspecified); J18.1 (Lobar pneumonia, unspecified); J18.2 (Hypostatic pneumonia, unspecified); J18.8 (Other pneumonia, organism unspecified); J18.9 (Pneumonia, unspecified)

ICD-10 CA, International Classification of Diseases, Tenth Revision (34).

Table S2. Annual incidence and case-fatality rates for hospitalizations due to all-cause pneumonia in Canada, 2004–2005 to 2009–2010 by age group

	2004–2005	2005–2006	2006–2007	2007–2008	2008–2009	2009–2010
**Annual incidence per 100,000 persons (95% CI)**						
0–4 years	694<br>(680, 708)	659<br>(646, 673)	638<br>(625, 652)	536<br>(524, 548)	562<br>(550, 575)	611<br>(598, 624)
5–9 years	125<br>(119, 131)	151<br>(145, 158)	124<br>(118, 130)	114<br>(108, 119)	104<br>(99, 110)	154<br>(148, 161)
10–14 years	48<br>(45, 52)	53<br>(50, 57)	46<br>(43, 49)	43<br>(40, 46)	37<br>(34, 40)	53<br>(50, 57)
15–19 years	40<br>(37, 43)	42<br>(39, 45)	39<br>(36, 42)	37<br>(34, 40)	35<br>(33, 38)	42<br>(39, 46)
20–29 years	46<br>(44, 49)	45<br>(43, 47)	45<br>(43, 48)	44<br>(42, 46)	41<br>(39, 44)	46<br>(44, 48)
30–39 years	76<br>(73, 79)	75<br>(72, 78)	75<br>(73, 78)	77<br>(74, 80)	66<br>(63, 69)	75<br>(72, 78)
40–49 years	111<br>(108, 115)	112<br>(109, 116)	114<br>(111, 118)	119<br>(116, 122)	111<br>(108, 114)	123<br>(120, 127)
50–59 years	222<br>(216, 227)	216<br>(211, 221)	212<br>(207, 217)	213<br>(208, 218)	210<br>(205, 215)	226<br>(221, 231)
60–69 years	549<br>(539, 559)	530<br>(520, 540)	515<br>(505, 525)	495<br>(486, 504)	500<br>(491, 509)	501<br>(492, 510)
70–79 years	1405<br>(1386, 1425)	1328<br>(1309, 1347)	1303<br>(1284, 1321)	1259<br>(1241, 1277)	1275<br>(1257, 1293)	1209<br>(1191, 1226)
≥80 years	3527<br>(3487, 3567)	3373<br>(3334, 3412)	3264<br>(3227, 3302)	3115<br>(3079, 3151)	3152<br>(3117, 3188)	3002<br>(2968, 3037)

	2004–2005	2005–2006	2006–2007	2007–2008	2008–2009	2009–2010
**Case-fatality, % (95% CI)**						
0–4 years	0.2<br>(0.1, 0.3)	0.2<br>(0.1, 0.4)	0.2<br>(0.1, 0.4)	0.2<br>(0.2, 0.4)	0.3<br>(0.2, 0.5)	0.2<br>(0.1, 0.3)
5–9 years	0.2<br>(0.1, 0.5)	0.3<br>(0.2, 0.7)	0.3<br>(0.1, 0.7)	0.3<br>(0.1, 0.7)	0.5<br>(0.2, 1.0)	0.4<br>(0.2, 0.8)
10–14 years	1.1<br>0.5, 2.2)	0.9<br>(0.5, 1.8)	0.7<br>(0.3, 1.6)	1.0<br>(0.5, 2.1)	1.2<br>(0.6, 2.5)	0.7<br>(0.3, 1.6)
15–19 years	2.1<br>(1.2, 3.5)	2.0<br>(1.2, 3.3)	0.9<br>(0.4, 2.0)	2.2<br>(1.3, 3.6)	1.1<br>(0.5, 2.3)	2.0<br>(1.2, 3.3)
20–29 years	2.1<br>(1.5, 2.9)	1.3<br>(0.8, 2.0)	1.9<br>(1.4, 2.8)	2.0<br>(1.4, 2.8)	2.7<br>(1.9, 3.6)	2.9<br>(2.2, 3.8)
30–39 years	3.2<br>(2.6, 4.0)	2.6<br>(2.0, 3.3)	2.5<br>(1.9, 3.1)	1.9<br>(1.5, 2.5)	2.3<br>(1.8, 3.0)	2.3<br>(1.8, 2.9)
40–49 years	5.3<br>(4.7, 6.0)	4.5<br>(3.9, 5.1)	4.3<br>(3.8, 4.9)	3.9<br>(3.4, 4.5)	4.3<br>(3.7, 4.9)	4.1<br>(3.6, 4.7)
50–59 years	7.8<br>(7.2, 8.4)	7.1<br>(6.5, 7.7)	7.5<br>(6.9, 8.1)	7.2<br>(6.7, 7.9)	6.8<br>(6.2, 7.3)	6.4<br>(5.9, 7.0)
60–69 years	10.4<br>(9.8, 11.0)	10.1<br>(9.5, 10.7)	9.9<br>(9.4, 10.5)	9.7<br>(9.1, 10.2)	10.1<br>(9.5, 10.6)	9.5<br>(9.0, 10.1)
70–79 years	14.0<br>(13.5, 14.5)	13.3<br>(12.8, 13.7)	13.3<br>(12.8, 13.8)	13.9<br>(13.4, 14.4)	13.6<br>(13.1, 14.1)	13.2<br>(12.7, 13.7)
≥80 years	20.5<br>(20.0, 20.9)	19.7<br>(19.3, 20.2)	20.9<br>(20.4, 21.3)	20.6<br>(20.1, 21.1)	20.4<br>(20.0, 20.9)	20.3<br>(19.8, 20.7)

CI, confidence interval.

Table S3. Annual incidence and case-fatality rates for hospitalizations due to pneumococcal pneumonia in Canada, 2004–2005 to 2009–2010 by age group

	2004–2005	2005–2006	2006–2007	2007–2008	2008–2009	2009–2010
**Annual incidence per 100,000 persons (95% CI)**						
0–4 years	10.64 (9.02, 12.54)	7.11 (5.81, 8.69)	7.96 (6.59, 9.61)	7.48 (6.17, 9.08)	7.21 (5.93, 8.76)	9.14 (7.70, 10.86)
5–9 years	1.62 (1.08, 2.41)	2.20 (1.55, 3.11)	1.40 (0.90, 2.17)	1.70 (1.14, 2.54)	1.28 (0.81, 2.03)	2.26 (1.60, 3.20)
10–14 years	0.85 (0.50, 1.43)	0.55 (0.29, 1.06)	0.68 (0.38, 1.23)	1.07 (0.66, 1.72)	0.26 (0.10, 0.68)	0.91 (0.54, 1.53)
15–19 years	0.53 (0.28, 1.03)	0.99 (0.62, 1.60)	1.15 (0.74, 1.79)	0.80 (0.47, 1.35)	0.86 (0.52, 1.42)	1.03 (0.65, 1.64)
20–29 years	1.54 (1.17, 2.03)	1.52 (1.15, 2.00)	2.11 (1.68, 2.66)	1.78 (1.39, 2.28)	1.32 (0.99, 1.76)	1.59 (1.23, 2.06)
30–39 years	3.12 (2.59, 3.75)	3.39 (2.82, 4.06)	3.64 (3.06, 4.33)	3.61 (3.03, 4.30)	2.74 (2.24, 3.35)	3.09 (2.56, 3.73)
40–49 years	4.48 (3.87, 5.18)	4.33 (3.73, 5.01)	5.97 (5.26, 6.76)	5.08 (4.43, 5.82)	4.07 (3.50, 4.74)	4.56 (3.95, 5.27)
50–59 years	7.27 (6.38, 8.28)	6.18 (5.38, 7.10)	5.94 (5.17, 6.82)	6.51 (5.71, 7.42)	5.30 (4.59, 6.12)	5.31 (4.61, 6.11)
60–69 years	13.78 (12.23, 15.52)	10.60 (9.27, 12.11)	10.43 (9.14, 11.91)	10.74 (9.46, 12.19)	9.56 (8.39, 10.90)	8.85 (7.75, 10.11)
70–79 years	19.63 (17.46, 22.08)	14.40 (12.56, 16.51)	14.83 (12.97, 16.95)	15.18 (13.30, 17.32)	13.25 (11.51, 15.25)	13.07 (11.36, 15.04)
≥80 years	30.83 (27.27, 34.86)	21.64 (18.74, 24.98)	20.57 (17.80, 23.76)	16.97 (14.51, 19.84)	17.37 (14.92, 20.23)	16.77 (14.40, 19.54)
**Case-fatality, % (95% CI)**						

	2004–2005	2005–2006	2006–2007	2007–2008	2008–2009	2009–2010
0–4 years	0.0	1.1 (0.1, 7.1)	0.0	0.0	0.0	0.8 (0.1, 5.3)
5–9 years	0.0	0.0	0.0	0.0	0.0	0.0
10–14 years	0.0	0.0	0.0	5.9 (0.8, 32.0)	25.0 (3.4, 76.2)	0.0
15–19 years	0.0	0.0	0.0	0.0	0.0	0.0
20–29 years	3.9 (1.0, 14.4)	2.0 (0.3, 12.6)	0.0	0.0	2.1 (0.3, 13.6)	1.7 (0.2, 11.2)
30–39 years	5.4 (2.4, 11.5)	3.4 (1.3, 8.6)	1.6 (0.4, 6.1)	2.4 (0.8, 7.1)	1.0 (0.1, 7.0)	3.7 (1.4, 9.4)
40–49 years	4.4 (2.2, 8.6)	2.8 (1.2, 6.6)	2.9 (1.4, 5.9)	1.4 (0.5, 4.4)	3.0 (1.3, 7.0)	4.3 (2.2, 8.4)
50–59 years	7.9 (5.1, 12.2)	9.0 (5.7, 13.8)	4.5 (2.4, 8.4)	5.4 (3.1, 9.2)	5.3 (2.9, 9.7)	3.6 (1.7, 7.4)
60–69 years	6.3 (3.9, 9.9)	5.1 (2.9, 9.0)	4.6 (2.5, 8.3)	6.3 (3.8, 10.1)	6.7 (4.1, 10.8)	7.8 (4.9, 12.2)
70–79 years	14.3 (10.7, 19.0)	10.2 (6.7, 15.1)	12.1 (8.4, 17.2)	10.0 (6.6, 14.7)	7.7 (4.7, 12.4)	8.7 (5.5, 13.6)
≥80 years	16.9 (12.8, 22.0)	18.3 (13.4, 24.5)	17.9 (13.0, 24.2)	19.1 (13.7, 26.0)	21.1 (15.5, 28.0)	17.6 (12.5, 24.2)

CI, confidence interval.
